# A New and Fast-Response Fluorescent Probe for Monitoring Hypochlorous Acid Derived from Myeloperoxidase

**DOI:** 10.3390/molecules28166055

**Published:** 2023-08-14

**Authors:** Małgorzata Świerczyńska, Daniel Słowiński, Radosław Michalski, Jarosław Romański, Radosław Podsiadły

**Affiliations:** 1 Institute of Polymer and Dye Technology, Faculty of Chemistry, Lodz University of Technology, Stefanowskiego 16, 90-537 Lodz, Poland; malgorzata.swierczynska@dokt.p.lodz.pl (M.Ś.); daniel.slowinski@dokt.p.lodz.pl (D.S.); 2 Institute of Applied Radiation Chemistry, Faculty of Chemistry, Lodz University of Technology, Zeromskiego 116, 90-924 Lodz, Poland; radoslaw.michalski@p.lodz.pl; 3 Department of Organic and Applied Chemistry, Faculty of Chemistry, University of Lodz, Tamka 12, 91-403 Lodz, Poland; jaroslaw.romanski@chemia.uni.lodz.pl

**Keywords:** fluorescent sulfur-containing probe, thiomorpholine-based probes, hypochlorous acid, stoichiometric reaction, myeloperoxidase

## Abstract

Hypochlorous acid (HOCl) has been implicated in numerous pathologies associated with an inflammatory component, but its selective and sensitive detection in biological settings remains a challenge. In this report, imaging of HOCl was realized with a thiomorpholine-based probe as derivative of nitrobenzothiadiazole (NBD-S-TM). The fluorescence is based on photoinduced electron transfer by using nitrobenzothiadiazole core as a donor and thiomorpholine substituent as an acceptor. NBD-S-TM showed high sensitivity and a fast response to HOCl *k* = (2.6 ± 0.2) × 10^7^ M^−1^s^−1^ with a 1:1 stoichiometry. The detection limit for HOCl was determined to be 60 nM. Furthermore, the desirable features of NBD-S-TM for the detection of HOCl in aqueous solutions, such as its reliability at physiological pH, rapid fluorescence response, and biocompatibility, enabled its application in the detection of HOCl in myeloperoxidase enzymatic system. Moreover, NBD-S-TM exhibited excellent selectivity and sensitivity for HOCl over other biologically relevant species, such as hydrogen peroxide and peroxynitrite. The fluorescent *S*-oxidized product (NBD-S-TSO) is only formed in the presence of HOCl. Probing with NBD-S-TM may be helpful to further the development of high throughput screening assays to monitor the activity of myeloperoxidase.

## 1. Introduction

**Figure 1:**
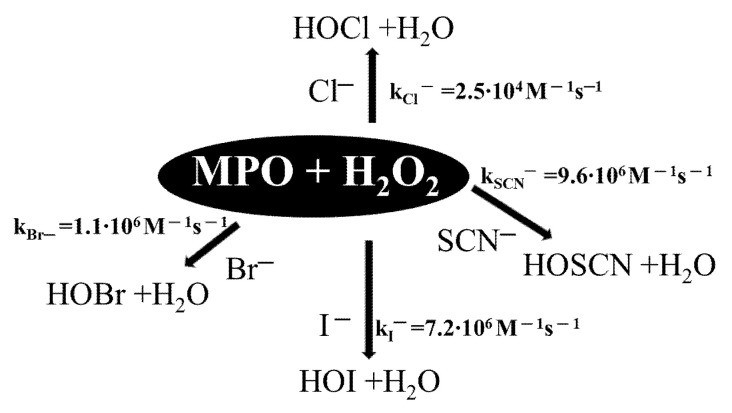
Catalyzed conversion of Cl^−^, Br^−^, I^−^, and SCN^−^ via MPO, with second-order constants of the reaction of Compound I with anions [2,5].

Myeloperoxidase (MPO) is an essential member of the heme peroxidase superfamily. This enzyme catalyzes the production of hypohalous acids (HOCl, hypochlorous acid; HOBr, hypobromous acid; HOI, hypoiodous acid) and also hypothiocyanous acid (HOSCN) from hydrogen peroxide (H_2_O_2_) and the respective halide ions (Cl^−^, Br^−^, I^−^), as well as thiocyanate ion (SCN^−^) (Figure 1) [1,2]. Hypochlorous acid is kinetically one of the most reactive species generated in vivo, exhibiting strong oxidizing and halogenating abilities [3,4].

Since the concentrations of chlorine anion are much higher (100–150 mM) than other halide/thiocyanate ions (Br^−^: 20–100 μM; I^−^: <0.1–1 μM, SCN^−^: 20–150 μM), Cl^−^ is a major physiological substrate for MPO [6]. Due to its high reactivity toward HOCl with biological targets and its limited selectivity, the excessive or misplaced production of HOCl can damage most biological molecules, with targets including proteins, DNA [7], RNA [8], and lipids [9]. Hypochlorous acid was found to react rapidly with proteins, being major targets of HOCl due to their high abundance and reactivity [10]. Hypochlorous acid reacts most rapidly with sulfur-containing amino acids (cysteine (Cys), methionine (Met), and cystine) [11]. Amine groups are also affected as less-reactive targets and the chloramines formed can induce protein cleavage and cross-linking [12,13]. As its reactivity with biomolecules is orders of magnitude higher than that of peroxynitrite (ONOO^–^) and H_2_O_2_, HOCl appears indispensable for fulfilling the host immune defense’s primary function: protecting the body against diseases [4]. The production of HOCl plays a crucial role in immune defense but also induces tissue damage [14]. It is closely related to many diseases, such as rheumatoid arthritis [15], neurodegenerative conditions [16], cardiovascular diseases [17], chronic kidney disease [18], lung and liver injury [19], and some cancers [9,20,21,22,23].

For the past few years, fluorescence probes have emerged as an ideal approach to hypochlorous acid detection due to their suitable properties [24]. Many fluorescent probes have recently been designed to monitor HOCl based on different recognition receptors [25,26,27,28]. Fluorescent probes have some unique advantages in many respects, and still, many lack the abilities for biological applications, e.g., low sensitivity, poor selectivity, and limited applications during in vivo bioimaging [29,30,31,32,33]. A major challenge in monitoring HOCl is that its precise roles in biological activities remain elusive. Therefore, developing new tools for HOCl detection with high selectivity, sensitivity, and accuracy is still in demand.

Among mechanisms, photoinduced electron transfer (PET) has attracted attention due to its “off-on” mode with a high signal-to-noise ratio [34]. Recently, we have shown that thiomorpholine effectively quenches the emission of the 4-nitro-benz-1,2,5-oxadiazole (NBD) and 7-nitrobenz-2-seleno-1,3-diazole fluorophore (NBD-Se), whereas the thiomorpholine *S*-oxide and thiomorpholine *S*,*S*-dioxide analogs are fluorescent [35,36]. This forced us to obtain pro-fluorescent thiomorpholine-based probes, which exhibit higher reactivity towards HOCl and react many times faster than the boronate and para-substituted aminophenyl probes, at least ten times faster than amines, and comparably fast with other biological thiols and thioethers [37]. However, NBD substituted with cyclic secondary amines, such as piperidine and piperazine, are known to be sensitive to hydrogen sulfide, and their fluorescence turns off under the influence of H_2_S under physiological. To ensure better stability of the thiomorpholine-based probe towards H_2_S, we decided to use nitrobenzothiadiazole (NBD-S) as a fluorophore due to the much lower reactivity of the piperazinyl- and piperidyl-substituted NBD-S compounds towards H_2_S [38].

The variety of donor–acceptor (D–A) combinations offers potential in the design and production of novel fluorescent materials that can also be used in optical and electrochemical applications [39,40,41]. Many D–A compounds are promising due to their stability and efficiency [42]. Benzothiadiazole is very often used as a building block of donor–acceptor materials with high electron affinity, which determines the energy of the lowest unoccupied molecular orbital. Its strong electron–acceptor properties provide unique properties, e.g., intramolecular and intermolecular interactions, charge transport properties, bandgap, etc. which can be controlled by various donors or π-coupled systems [43].

Based on these advantages, we synthesized an NBD-S derived probe (NBD-S-TM) with a thiomorpholine moiety as a receptor, in which sulfur is a chlorination site via HOCl. Next, the chlorosulfonium ion hydrolyzed to the fluorescent sulfoxide [44]. As a result, NBD-S-TM responded to HOCl with excellent selectivity, high sensitivity, and fast response. Furthermore, compared to the other reported HOCl probes, according to the literature, NBD-S-TM may have exceptional lysosomal targeting ability and be suitable for lysosomal HOCl detection due to its morpholino-derived moiety (lysosome locating group) [45,46]. Moreover, NBD-S-TM was also successfully applied to detect HOCl in the enzyme system, indicating our bioimaging strategy’s feasibility.

## 2. Results and Discussion

### 2.1. Molecular Design and Synthesis

**Scheme 1:**
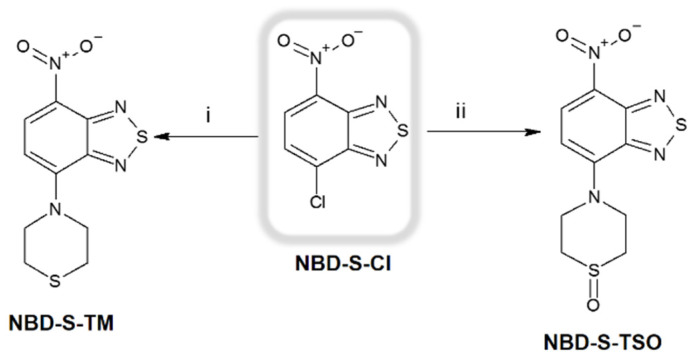
Synthetic route for NBD-S-TM and NBD-S-TSO. Reagents and conditions: (i) thiomorpholine, Et_3_N, and DCM at room temperature for 20 h under an argon atmosphere; (ii) thiomorpholine *S*-oxide, Et_3_N, and DCM at room temperature for 20 h under an argon atmosphere.

From a biological point of view, HOCl derived from myeloperoxidase can participate in non-enzymatic reactions, e.g., oxidation and chlorination of cellular components. The reaction rate constant is a parameter that controls and determines the performance of the probe in the detection of MPO chlorinating activity. Recently, we have reported that the thiomorpholine-based NBD-TM probe and its selenium analog scavenge HOCl with the rate constants of 1 × 10^7^ and 2 × 10^7^ M^−1^s^−1^, respectively [35,37]. Here, we design the NBD-S-TM probe with improved stability for fluorescent HOCl detection. We also synthesized its sulfoxide, NBD-S-TSO, the product formed in the reaction between NBD-S-TM and HOCl. Both nitrobenzothiadiazole-containing derivatives (NBD-S-TM and NBD-S-TSO) were obtained in good yields via a straightforward synthetic protocol (Scheme 1). NMR and HRMS analyses confirmed the chemical structure of the compounds (see Supplementary Materials).

### 2.2. Spectroscopic Characterization of the Probe and its Oxidation Product

**Figure 2:**
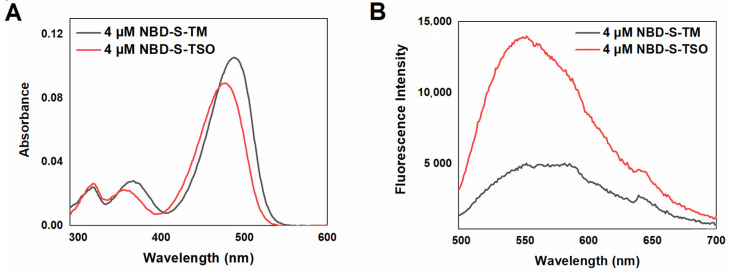
Spectroscopic characterization of NBD-S-TM and NBD-S-TSO. (**A**) Electronic absorption and (**B**) fluorescence spectra of nitrobenzothiadiazole derivatives in phosphate buffer (50 mM, pH 7.4). The concentration of the relevant compound was 4 µM in each case. λ_ex_ = 490 nm, ex/em slit: 2.5 nm.

The spectroscopic properties of nitrobenzothiadiazoles are presented in Table 1. Absorption and fluorescence spectra at pH of 7.4 are shown in Figure 2. The probe and the *S*-oxide carrying the nitrobenzothiadiazole scaffold have absorption bands in the visible light range located at 400–550 nm. The presence of the thiomorpholine *S*-oxide group causes/caused a little blue shift (15 nm) of the NBD-S-TSO absorption band compared to non-oxidized NBD-S-TM. A similar small hypsochromic effect (15–20 nm) was observed for the 4-thiomorpholino-7-nitrobenz-2-oxa-1,3-diazole (NBD-TM) and [35] 4-thiomorpholine-7-nitrobenz-2-seleno-1,3-diazole (NBD-Se-TM) and their *S*-oxide [36]. The fluorescence spectra of NBD-S-TM and NBD-S-TSO (Figure 2B) show similar fluorescence maximum located at 550 nm. The strong electron-donating substituent (thiomorpholine oxide) vastly increases the fluorescence intensity of NBD-S-TSO compared to the NBD-S-TM probe. Compared to other *S*-oxides (NBD-TSO and NBD-Se-TSO), the NBD-S-TSO compound is characterized by the highest emission quantum efficiency, and more importantly, this *S*-oxide has a higher emission quantum yield than the NBD-S-TM probe. This indicates the possibility of fluorescent detection of HOCl under physiological pH conditions.

### 2.3. Spectral Response of NBD-S-TM to HOCl

**Figure 3:**
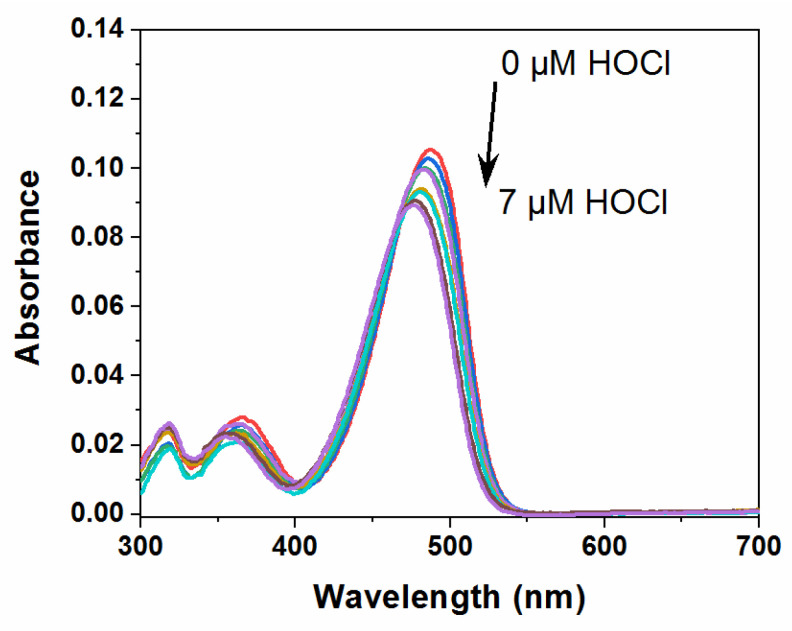
Electronic absorption spectra of NBD-S-TM (4 µM) upon titration of HOCl (0–7 µM) in a phosphate buffer solution (50 mM, pH 7.4) with MeCN (10%).

**Figure 4:**
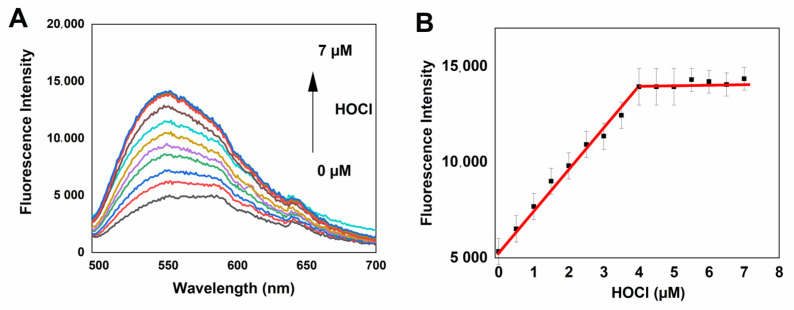
(**A**) Fluorescence spectra of NBD-S-TM (4 µM) at different HOCl content (0–7 µM) in a phosphate buffer solution (50 mM, pH 7.4) with MeCN (10%); λ_ex_ = 490 nm, λ_em_ = 550 nm).; (**B**) The plot of the intensity at 550 nm versus the concentration of HOCl (0–7 µM).

To investigate the performance of the NBD-S-TM probe with HOCl, we conducted spectral studies in phosphate buffer (50 mM, pH = 7.4) containing 10% of acetonitrile. We chose MeCN as co-solvent instead of dimethyl sulfoxide due to this solvent tremendously impacting the on-detected HOCl concentrations. The absorption spectra of the NBD-S-TM probe recorded before and after the bolus addition of HOCl are shown in Figure 3. Upon adding HOCl, the absorbance at 490 nm gradually decreased, and a blue shift of the maximum absorption band from 490 nm to 475 nm was observed. Next, we investigated a fluorescence response of the NBD-S-TM probe towards HOCl and as shown in Figure 4A, the fluorescence intensity at 550 nm increased upon adding HOCl, as well as the color of the solution under 365 nm UV light changed from colorless to yellow-green. When 4 µM HOCl was added, an approximate 3-fold fluorescence enhancement was observed (Figure 4A).

At that time, the fluorescence intensity reached a maximum value, but the fluorescence intensity remained almost unchanged with a further increase in HOCl. Based on the fluorescence titration data, the fluorescence intensity plot at different HOCl concentrations was made (Figure 4B), and the linear relationship (R = 0.995) for HOCl concentrations in the range of 0–4 µM was observed.

Based on the 3r/k (r—standard deviation of blanks; k—slope of titration curve), the detection limit was 60 nM, meaning that the probe NBD-S-TM possessed the highest sensitivity to HOCl compared to previously reported 4-thiomorpholine-7-nitrobenzoxadiazole (NBD-TM) and 4-thiomorpholine-7-nitrobenzoselenadiazole (NBD-Se-TM) (Table 2).

### 2.4. HPLC Titration

**Figure 5:**
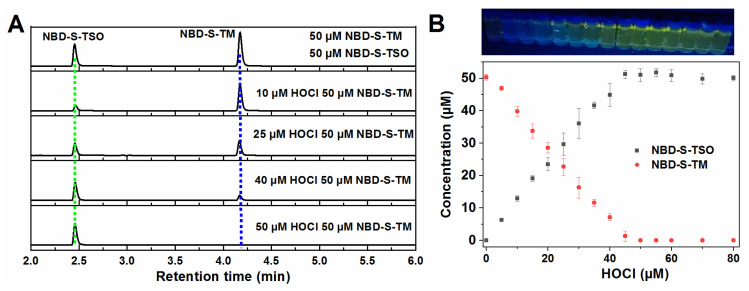
HOCl-induced oxidation of the NBD-S-TM probe. (**A**) The chromatograms of NBD-S-TM, NBD-S-TSO (50 μM each), and a mixture of NBD-S-TM (50 μM) with HOCl (0, 10, 25, 40, and 50 μM) in a phosphate buffer (50 mM, pH 7.4) with MeCN (10%). (**B**) HPLC-based titration. Dependence of the disappearance/formation of NBD-S-TM/NBD-S-TSO on the amount of HOCl. Incubation mixtures comprised 50 µM NBD-S-TM and 0–80 µM of HOCl in phosphate buffer (50 mM, pH 7.4). NBD-S-TM and its corresponding oxidation product were detected using absorption detection at 500 nm. Data are means ± standard deviation of three independent experiments. The HPLC traces were collected after 5-min incubation of NBD-S-TM with oxidant using the absorption detector set at 500 nm. Inset: Photograph of quartz cuvettes containing the NBD-S-TM probe with different amounts of HOCl under UV light (365 nm).

**Figure 6:**
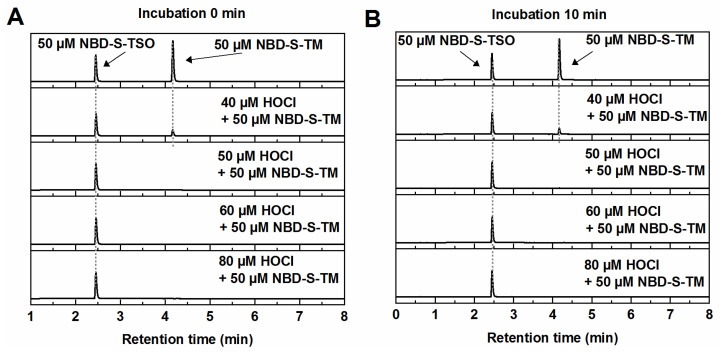
Stability of NBD-S-TSO in the presence of HOCl excess. HPLC traces of the products detected during the reaction between NBD-S-TM (50 µM) and HOCl (40 µM, 50 µM, 60 µM, 80 µM) in phosphate buffer (50 mM, pH 7.4) with MeCN (10%) at 25 °C. (**A**) after 0-min incubation; (**B**) after 10-min incubation. The HPLC traces were collected using the absorption detector set at 500 nm.

The reaction between NBD-S-TM and HOCl does not shift the solution’s fluorescence, with the product’s spectrum resembling that of the authentic spectrum of NBD-S-TSO (Figure 4). However, there is a distinct linear increase in fluorescence intensity at 550 nm with successive addition of HOCl. A further rise in the HOCl concentration above the probe concentration does not cause any changes in the fluorescence spectrum, and neither the intensity signals nor the bands emission position changes. This suggests a 1:1 reaction stoichiometry between the NBD-S-TM probe and HOCl (Figure 4). To confirm the stoichiometry reaction between the probe and hypochlorous acid, high-performance liquid chromatography (HPLC) was used to separate and detect the products formed in the HOCl-induced oxidation of NBD-S-TM. As shown in Figure 5, NBD-S-TM and NBD-S-TSO have retention times at 4.28 and 2.48 min, respectively. After adding HOCl to NBD-S-TM, the characteristic peak of S-oxide appeared after 2.48 min, and the intensity of the peak was correlated with the increase in the concentration of HOCl, at which the concentration of NBD-S-TM in the sample decreased accordingly (Figure 5). Chromatograms (Figure 5A and Figure 6) also confirmed that NBD-S-TSO was the sole product of the reaction of NBD-S-TM with HOCl, even when the oxidant was present in excess to the probe. Moreover, we did not observe further oxidation or degradation of the NBD-S-TSO throughout experimentation, even after 10 min incubation with an excess of the oxidant.

Titration of the probe with HOCl results in the product profile shown in Figure 5B, indicating the formation of the thiomorpholine *S*-oxide in a dose-dependent manner. The highest yield of the NBD-S-TSO product was close to 98%, and a slight excess of the oxidant was required for the complete consumption of the NBD-S-TM probe. These data agreed with the fluorescence measurements conducted under the same experimental conditions (Figure 4).

**Scheme 2:**
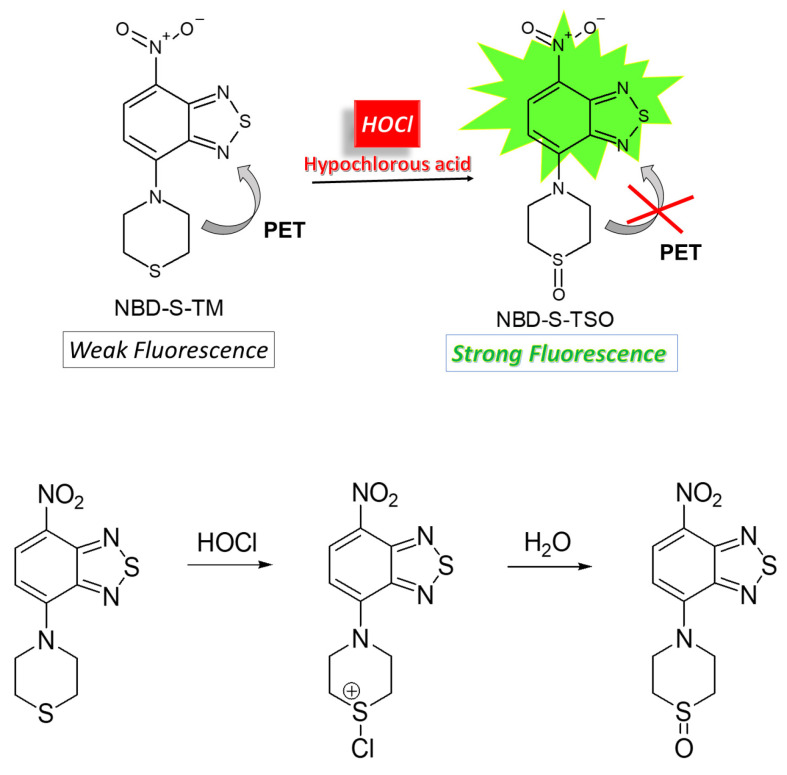
Proposed mechanism of HOCl-induced oxidation of NBD-S-TM.

Based on conducted experiments and previous literature reports [35,36], we conclude that HOCl oxidizes the NBD-S-TM probe to the NBD-S-TSO according to the proposed mechanism presented in Scheme 2. Without adding HOCl, the fluorescence probe was quenched due to the PET effect. The fluorescence intensity at 550 nm increased by 3-fold after HOCl was added, which can be attributed to the formation of NBD-S-TSO.

### 2.5. Stability and Selectivity Studies

**Figure 7:**
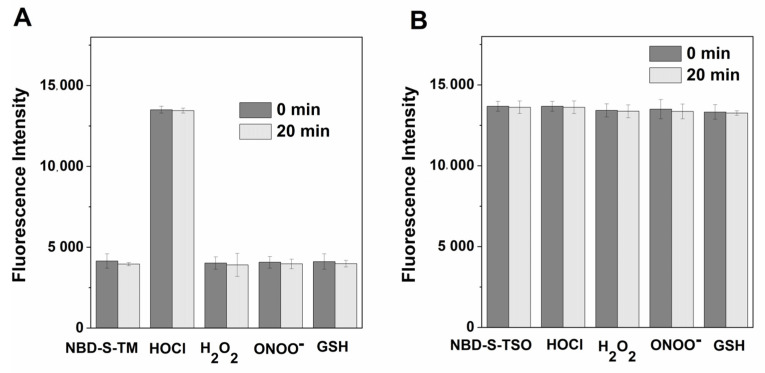
Fluorescence responses at 550 nm in phosphate buffer (50 mM, pH 7.4) after the addition of HOCl, H_2_O_2_, ONOO^−^, and GSH (4 μM, each) at various times of incubation (0 and 20 min). (**A**) NBD-TM (4 μM); (**B**) NBD-S-TSO (4 µM). Data are means ± standard deviation of three independent experiments.

An essential challenge for the development of reliable HOCl detection methods and probes to monitor small changes at the biological level remains to develop highly selective and sensitive probes characterized by a main and specific reaction with HOCl compared to other oxidants, e.g., peroxynitrite or hydrogen peroxide. The limitation of known boronate-based in vivo sensors is their lack of specificity [48]. In addition, the detection of HOCl in vivo is complicated as the existence of various antioxidants, such as GSH [49,50] and compounds present in the cellular environment at high concentrations [3,51]. Several reports [52,53,54,55,56,57] described that sulfur- and selenium-oxides formed in reaction with HOCl are reduced via GSH to a sulfur and selenide group, respectively. Reactions with GSH may also result in the formation of an adduct with GSH, which reduces fluorescence probe response [58]. The fluorescence response of both the NBD-S-TM probe and NBD-S-TSO to GSH, H_2_O_2_, and ONOO^–^ was analyzed, and the effect of incubation time in the presence of all three reactants was studied. Figure 7 shows no noticeable fluorescence intensity changes of the probe and its sulfoxide in the presence of GSH, H_2_O_2_, and ONOO^–^. Moreover, the fluorescence signals were constant even with the extended incubation time of up to 20 min.

**Figure 8:**
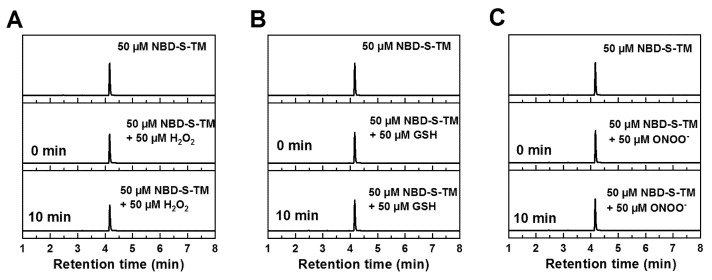
The reaction between NBD-S-TM and oxidants/GSH in an aqueous solution containing phosphate buffer (50 mM, pH 7.4) and MeCN (10%) after 0- and 10-min incubation at 25 °C. HPLC chromatogram of NBD-S-TM (50 μM) and the reaction mixture of NBD-S-TM (50 μM) with (**A**) H_2_O_2_ (50 μM); (**B**) GSH (50 μM); (**C**) ONOO^–^ (50 μM). The traces were collected using an absorption detector set at 500 nm.

**Figure 9:**
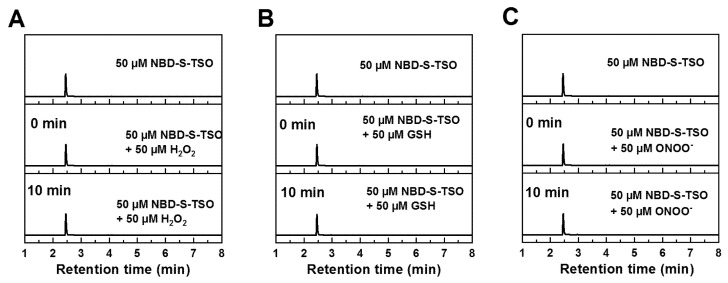
The reaction between NBD-S-TSO and oxidants/GSH in an aqueous solution containing phosphate buffer (50 mM, pH 7.4) and MeCN (10%) after 0- and 10-min incubation at 25 °C. HPLC chromatogram of NBD-S-TSO (50 μM) and the reaction mixture of NBD-S-TSO (50 μM) with (**A**) H_2_O_2_ (50 μM); (**B**) GSH (50 μM); (**C**) ONOO^–^ (50 μM). The traces were collected using an absorption detector set at 500 nm.

Furthermore, we carried out HPLC analyses to check the stability of the NBD-S-TM probe and the NBD-S-TSO after the addition of GSH, H_2_O_2_, and ONOO^−^. HPLC chromatograms displayed in Figure 8 and Figure 9 indicate that there were no formation of new product(s). Moreover, NBD-S-TM and NBD-S-TSO were stable over 10-min incubation periods, which confirm the previous results obtained from HPLC measurements (Figure 8 and Figure 9).

**Figure 10:**
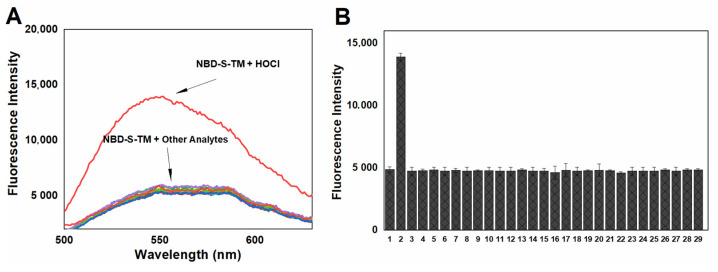
The selectivity of NBD-S-TM. (**A**) Fluorescence spectra of NBD-S-TM (4 μM) in the presence of HOCl (4 μM) and various analytes (4 μM). (**B**) Fluorescence responses of NBD-S-TM (4 μM) to various analytes (4 μM). Analytes: 1. probe, 2. HOCl, 3. H_2_O_2_, 4. ONOO^–^, 5. GSH, 6. Na^+^, 7. K^+^, 8. Li^+^, 9. Ag^+^, 10. Mg^2+^, 11. Ca^2+^, 12. NH_4_^+^, 13. Cu^2+^, 14. Zn^2+^, 15. Fe^3+^, 16. Hg^2+^, 17. Cl^–^, 18. I^–^, 19. HCO_3_^–^, 20. NO_2_^–^, 21. NO_3_^–^, 22. HS^–^, 23. HSO_3_^−^, 24. SO_4_^2-^, 25. S_2_O_8_^2-^, 26. CH_3_COO^–^, 27. TBHP, 28. NO^•^, 29. HNO. Data are means (±) standard deviation of three independent experiments. All experiments were conducted in an aqueous solution containing phosphate buffer (50 mM, pH 7.4) with MeCN (10%) at room temperature; λex = 490 nm, λem = 550 nm.

To check the potential application of the probe in a more complex system, the NBD-S-TM fluorescence response to various substrates was explored. Compared with HOCl, the variety of analytes/biological substances did not induce noticeable fluorescence changes at 550 nm (Figure 10), demonstrating that NBD-S-TM possessed high selectivity towards hypochlorous acid. As depicted in Figure 10A, the addition of HOCl to the solution of NBD-S-TM can significantly enhance the fluorescence intensity. However, the addition of various ions and some oxidants to NBD-S-TM solutions did not show any significant changes in fluorescence intensity. As shown in Figure 10B, a 3-fold fluorescence intensity enhancement was observed for NBD-S-TM after incubation with one equivalent of HOCl. In contrast, its fluorescence change was negligible in the presence of an equal equivalent (4 µM) of other ROS (e.g., H_2_O_2_, ONOO^–^), reducing agents (GSH, HS^–^), or redox-sensitive metal ions (Fe^3+^). Therefore, probe NBD-S-TM showed outstanding selectivity, and is a powerful tool for the specific detection of endogenous HOCl in complex physiological environments.

**Figure 11:**
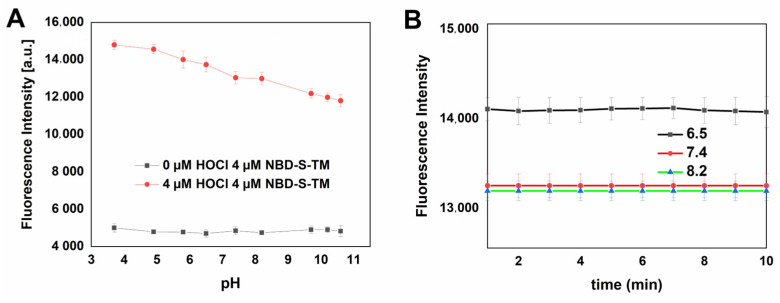
(**A**) The fluorescence emission intensity of NBD-S-TM (4 μM) in the presence or absence of HOCl (4 μM) in phosphate buffer solutions with different pH values. (**B**) The fluorescent response of the probe (4 μM) to the equimolar concentration of HOCl (4 μM) at different pH (6.5–8.2). All experiments were conducted in an aqueous solution containing phosphate buffer (50 mM, pH 7.4) with MeCN (10%) at room temperature; λ_ex_ = 490 nm, λ_em_ = 550 nm. Data are means ± standard deviation of three independent experiments.

Finally, the stability of NBD-S-TM and its response-ability to HOCl in various pH were tested. As depicted in Figure 11A, without the addition of HOCl, the fluorescence intensity of NBD-S-TM remained almost unchanged at the pH range (3.6–10.8). However, the fluorescent response of the probe towards HOCl slightly depended on the pH of the solution and decreased with increasing pH. Our experiments validate that the probe is proficient across a broad pH range and can be utilized in physiological pH conditions. Moreover, the fluorescence signal intensity at pH 6.5, 7.4, and 8.2 was also constant during the extended incubation time as depicted in Figure 11B.

### 2.6. Oxidation of NBD-S-TM via the MPO/H_2_O_2_ System

**Figure 12:**
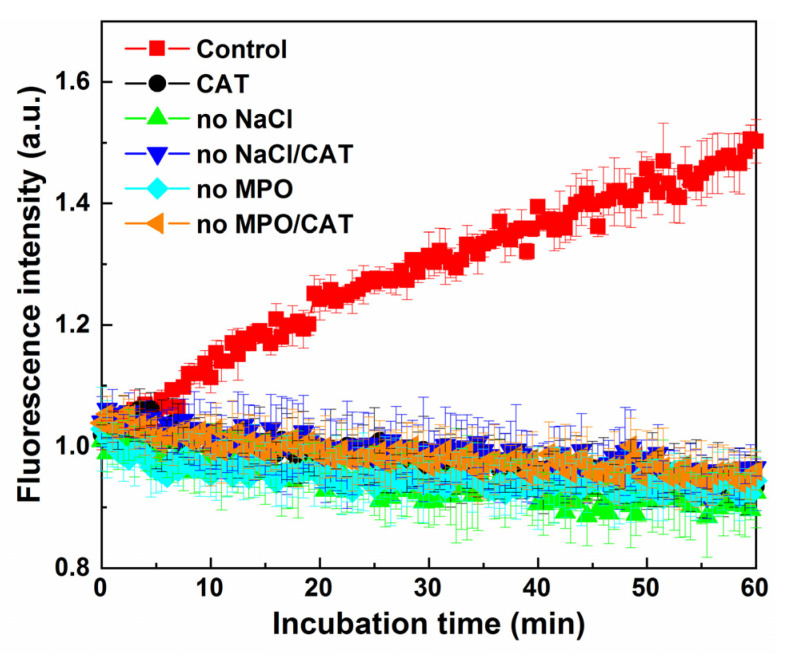
Continuous monitoring of MPO-derived HOCl production via monitoring the increase in NBD-S-TSO fluorescence intensity in the incubation mixtures containing NBD-S-TM. Compositions of incubation mixtures are in accordance with the figure legend. Concentration of the incubation mixture components was 0.9 nM MPO, 10 μM H_2_O_2_, 0.1 M NaCl, and 50 mM phosphate buffer (pH 7.4). Data are means ± standard deviation of three independent experiments.

The monitoring of HOCl formation in the enzyme system was determined using the NBD-S-TM probe, thus determining its efficiency. The enzyme system consisted of the NBD-S-TM probe incubated in phosphate buffer at pH 7.4 with the addition of 0.9 nM MPO, 0.1 M NaCl, and 10 μM H_2_O_2_. An increase in fluorescence intensity was observed for this incubation mixture (Figure 12). The presence of catalase or the absence of chloride anions or MPO in the incubation mixture resulted in no increase in fluorescence intensity. An enzyme incubation mixture containing all necessary ingredients to generate HOCl oxidized the NBD-S-TM probe to NBD-S-TSO. Studies confirm that the probe is suitable for monitoring the formation of HOCl analyte in MPO catalyzed enzymatic reactions.

### 2.7. Kinetic Measurements

**Figure 13:**
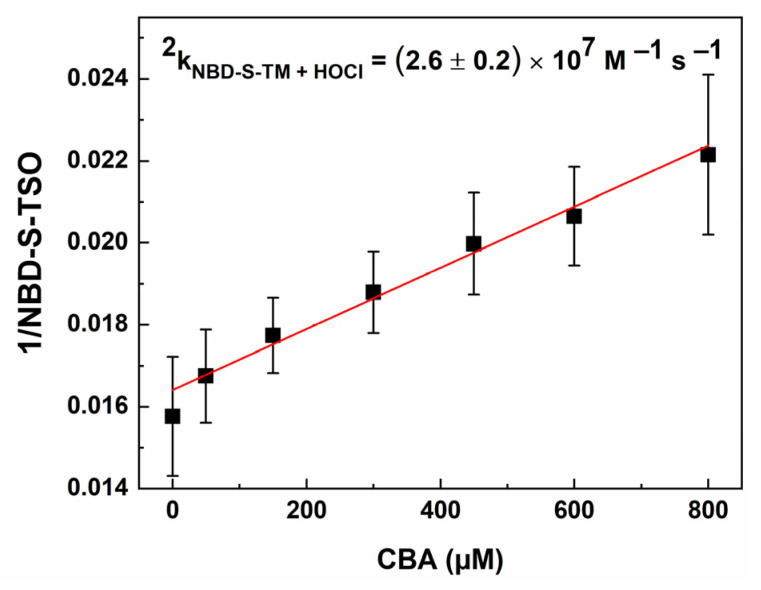
Kinetic of the reaction of NBD-S-TM with HOCl. The dependence of 1/[NBD-S-TSO] on the CBA concentration used to determine the reaction rate constant of NBD-S-TM with HOCl. Incubation mixtures containing 0–800 μM CBA, 1.5 μM NBD-S-TM, 0.5 µM HOCl, phosphate buffer (20 mM, pH 7.4), and 10% (*v*/*v*) ACN.

Fluorescent probes are becoming increasingly important tools used in the detection and imaging of biological signaling molecules due to their simplicity, high selectivity, and sensitivity, as well as non-invasiveness and suitability for real-time analysis of living systems. One of the important parameters in the development of fluorescent probes for the detection of selected reactive compounds in a biological environment is the rate of their reaction with it [59,60,61]. Detection of HOCl, especially in aqueous solutions, where it shows high stability, is not difficult. Only in the presence of biologically relevant molecules such as thiols or amines, is the lifetime of HOCl short. The main scavengers of HOCl are thiols and thioethers, of which reaction rate constants with HOCl range from 10^7^ to 10^8^ M^−1^s^−1^. The HOCl reaction rate constants for amines cover several orders of magnitude (10^1^–10^6^ M^−1^s^−1^) [59,62,63,64,65]. To investigate whether the NBD-S-TM probe can efficiently scavenge HOCl, we determined the second-order reaction rate constant of this reaction (Figure 13). Using competition kinetics to find the rate constant of HOCl with NBD-S-TM, we used coumaric boric acid (CBA) as the reference compound. The obtained second-order rate constant (2.6 ± 0.2) × 10^7^ M^−1^s^−1^ is in reasonable agreement with the rate constants determined for its structural counterparts NBD-TM (^2^k 1.0 × 10^7^ M^−1^s^−1^) and NBD-Se-TM (^2^k = 2.0 ± 0.2 10^7^ M^−1^s^−1^) [35,36] and other thioethers such as L-methionine (^2^k = 3.4 × 10^7^ M^−1^s^−1^) [37]. Studies confirm that NBD-S-TM can be used in the detection of HOCl in intracellular and extracellular environments because the rate constant obtained is comparable to that of HOCl in reaction with thiols and thioethers, and an order of magnitude faster than amines.

## 3. Materials and Methods

### 3.1. Materials, Instruments, and Methods

4-chloro-7-nitro-2,1,3-benzothiadiazole (NBD-S-Cl) and thiomorpholine (TM) were purchased from Merck. Myeloperoxidase (MPO) was from Athens Research and Technology (Athens, Georgia). Hydrogen peroxide (H_2_O_2_) and hypochlorous acid (HOCl) were purchased from Merck. Catalase (CAT, from bovine liver) was received from Sigma–Aldrich (St. Louis, MO, USA). Peroxynitrite was prepared as reported previously and stored at −80 °C [66]. For thin-layer chromatography (TLC), pre-coated aluminum-backed plates (Merck Kieselgel 60 F_254_) were used. Column chromatography purifications were performed on Merck Silica gel 60 (70–230 mesh). Other reagents and starting materials for chemical syntheses were purchased from commercial vendors and used without further purification. All the solvents were dried according to the standard methods before use. All solvents were either HPLC or spectroscopic grade in the optical spectroscopic studies. The purity of the NBD-S-derived compounds was checked via high-performance liquid chromatography (HPLC) on a Shimadzu UFLC system equipped with a diode array detector. The structures were confirmed by ^1^H, and ^13^C NMR spectra were acquired on a Bruker Avance III 600 spectrometer and were referenced according to the residual peak of the DMSO-*d*_6_ based on the literature data. Chemical shifts (δ) were quoted in ppm and coupling constants (J) in Hz. Solutions were prepared in DMSO-*d*_6_ with 20% of CDCl_3_. For high-resolution mass spectrometry (HRMS) studies, a Synapt G2-Si mass spectrometer (Waters) was used, which was equipped with an electrospray ionization (ESI) source and a quadrupole time-of-flight (QToF) mass analyzer. The capillary voltage was set to 2.7 kV, while the sampling cone voltage was set to 20 V. The source temperature was set to 110 °C. MassLynx 4.1 (Waters) software was used to process the results.

### 3.2. Synthesis

The syntheses of NBD-S-TM and NBD-S-TSO are depicted in Scheme 1, and 4-thiomorpholine-7-nitrobenzothiadiazole (NBD-S-TM) was synthesized by aromatic nucleophilic substitution by thiomorpholine at position C-7 in nitrobenzothiadiazole (NBD-S-Cl). The progress of the process was monitored by HPLC to verify competition of the reaction. In the synthesis of NBD-S-TSO, thiomorpholine *S*-oxide was utilized according to the same procedure. Detailed synthetic procedures for all compounds are shown in the Supplementary Materials.

### 3.3. Solutions Preparation

For all experiments, stock solutions of the NBD-S-TM probe and the *S*-oxide analog (NBD-S-TSO) were prepared by dissolving the solid compounds in acetonitrile (MeCN, probe/standard concentration: 1 mM). The mixture of HOCl or ONOO^–^ (concentration: 1 mM) was prepared in 0.1 M NaOH. The concentration of HOCl was determined by spectrophotometry, using the extinction coefficient value of 350 M^–1^cm^–1^ (at 292 nm, in 0.1 M NaOH). The concentration of ONOO^−^ was determined by spectrophotometry, using the extinction coefficient value 1.7 × 10^3^ M^−1^cm^−1^ (at 302 nm, in 0.1 M NaOH). The hydrogen peroxide was prepared in water (H_2_O, H_2_O_2_ concentration: 1 mM) and was also determined via spectrophotometry, using the extinction coefficient value of 39.4 M^−1^cm^−1^ (at 240 nm, in water). The working solutions of ONOO^−^, HOCl, and H_2_O_2_ were freshly prepared before each experiment and kept on ice [66]. The phosphate buffer was prepared from monosodium phosphate, monohydrate (0.1 M), and its conjugate base, disodium phosphate (0.1 M). Solutions of oxidants were added directly to the buffered reaction mixtures. All measurements were conducted in a phosphate buffer (50 mM, pH 7.4) containing MeCN (10%). To investigate selectivity towards ions, various salt stock solutions (1 mM; Na^+^, K^+^, Li^+^, Ag^+^, Mg^2+^, Ca^2+^, NH_4_^+^, Cu^2+^, Zn^2+^, Fe^3+^, Hg^2+^, Cl^–^, I^–^, HCO_3_^–^, NO_2_^–^, NO_3_^–^, HS^–^, HSO_3_^–^, SO_4_^2-^, S_2_O_8_^2-^, ^2^, CH_3_COO^–^) were freshly prepared by dissolving weighed portions of the corresponding salts in deionized water. The stock solution of glutathione (GSH) was freshly prepared in distilled water at concentrations of 1 mM. (Z)-1-[N-[3-aminopropyl]-N-[4-(3-aminopropylammonio)butyl]-amino]diazen-1-ium-1,2-diolate (Spermine NONOate) was employed as a source of nitrogen oxide (NO^•^). The nitrogen oxide fluxes were determined from the donor’s decomposition rate and measured following the decrease in its characteristic absorbance at 250 nm (ε = 8.5 × 10^3^ M^−1^cm^−1^). At 25 °C, in solutions containing a phosphate buffer (pH 7.4, 50 mM), 60 μM of spermine NONOate produced 0.72 μM/min NO^•^. Nitroxyl (HNO) was generated from Angeli’s salt (0.1 mM) prepared in NaOH (0.1 M). HNO flux was determined spectrophotometrically at λ_max_ = 248 nm using ε = 8.3 × 10^3^ M^−1^cm^−1^. tert-Butyl hydroperoxide (TBHP) (70 wt % in H_2_O) was freshly diluted to a concentration of 5 mM in H_2_O and immediately added to the NBD-S-TM-tested solution in phosphate buffer containing MeCN (H_2_O, TBHP concentration: 1 mM). All analytes were rapidly mixed with NBD-S-TM using a vortex mixer (5 s) before HPLC and spectroscopic analyses. All experiments were performed with the addition of acetonitrile (MeCN) at up to 10%.

### 3.4. Spectral Measurements

The absorption spectra were recorded on a Jasco V-670 UV-Vis-NIR spectrophotometer (Jasco, Tokyo, Japan). Steady-state fluorescence spectra were recorded using an FLS 920 fluorescence spectrophotometer (Edinburgh Instrument Ltd., Livingston, UK). The stock solution of NBD-S-TM (1 mM) was prepared in HPLC grade MeCN. Stock solutions of analytes were prepared in distilled water. The NBD-S-TM probe was diluted to 4 µM with 50 mM phosphate buffer solution (pH 7.4, 10% MeCN) for spectral measurements. Next, the solution (3.0 mL) was placed in a quartz cell of 1 cm optical path length each time. All spectroscopic experiments were carried out at room temperature. The excitation wavelength was 490 nm. Excitation and emission slit widths were 2.5 nm and 2.5 nm, respectively.

### 3.5. HPLC Analyses

HPLC analyses of NBD-S-TM and its oxidation product (NBD-S-TSO) were performed using a Shimadzu instrument (Kyoto, Japan). The samples (50 μL) were separated using a Kinetex C18 column (Phenomenex, 100 mm × 4.6 mm, 2.6 μm), which equilibrated 0.1% trifluoroacetic acid aqueous solution containing 10% MeCN. Gradient elution was performed at a 1.5 mL/min flow rate with absorption detection (λ = 500 nm). The compounds were eluted by raising MeCN concentration from 10% to 100% over 9 min. Under these conditions, NBD-S-TM shows a retention time of 4.28 min and NBD-S-TSO of 2.48 min. In a typical experiment, the organic solvent acetonitrile (MeCN, concentration: 1 mM) was used to prepare stock solutions of NBD-S-TM and NBD-S-TSO. To obtain a final freshly diluted concentration of 50 mM sample/standard, the appropriate amount of stock solutions was added to an aqueous solution of phosphate buffer (50 mM, pH 7.4) with 10% MeCN. The NBD-S-TM probe and all products formed in the reaction with HOCl were detected by monitoring the absorbance at 500 nm. In HPLC-based titration experiments to the NBD-S-TM (50 μM) solution in buffer/MeCN, the appropriate amount of HOCl stock solution (0–140 μL) was added. Alternatively, in the case of competition kinetic experiment, the compounds were eluted by raising MeCN concentration from 20% to 50% over 6 min and the fluorescent NBD-S-TSO was detected using excitation wavelength of 490 nm and emission wavelength of 550 nm.

### 3.6. Plate Reader Measurements

The Thermo Scientific Varioskan LUX multimode microplate reader was used in the study by monitoring changes in fluorescence intensity (λex = 490 nm, λem = 550 nm and slit width 12 nm). Enzymatic processes were monitored with human neutrophil myeloperoxidase. The enzyme stock solution was prepared according to the supplier’s instructions. To obtain the necessary MPO activity (5 nM/sec on a plate), the stock solution was diluted with phosphate buffer (50 mM, pH 7.4). Then, aliquot of the diluted enzyme solution was directly pipetted into a black flat-bottomed 96-well plate. 100 μL of freshly prepared solutions containing 20 μM probe and, depending on the control, 200 U/mL catalase and/or 0.2 M NaCl were added to each well of the microplates. The reaction was started by adding of 100 μL per well of 20 µM H_2_O_2_ solution.

### 3.7. Kinetic Study

The kinetics of the oxidation of NBD-S-TM by HOCl were studied under pseudo-first order conditions. The reaction rate constant of NBD-S-TM with HOCl was determined by a competition kinetics experiment using the CBA boronate probe as competitor and the reported rate constant of ^2^k_CBA+HOCl_ = 1.8 × 10^4^ M^−1^s^−1^ [37]. A set of incubation mixtures containing NBD-S-TM (1.5 μM) and CBA (0–800 μM) was prepared. Next, HOCl was added, and the concentration of NBD-S-TSO formed was determined by HPLC. The following equation was used when two probes, CBA and NBD-S-TM, simultaneously competed for the oxidant:

$$\frac{1}{\left[NBD-S-TSO\right]}=\frac{1}{{\left[NBD-S-TSO\right]}_{0}}+\frac{1}{{\left[NBD-S-TSO\right]}_{0}}\;\frac{k_{CBA+HOCl}\;\left[CBA\right]}{k_{NBD-S-TM+HOCl}\;\left[NBD-S-TM\right]}$$

From the plot of the reciprocal peak area of NBD-S-TSO versus the concentration of the reference compound (CBA) the second-order rate constant was determined.

## 4. Conclusions

In summary, the synthesis and the detailed spectroscopic and chemical characterization of a novel thiomorpholine-based fluorescent probe are described. 4-Thiomorpholine-7-nitrobenzothiadiazole (NBD-S-TM) was synthesized via simple one-step methodology under standard laboratory conditions and provided a good overall yield and high purity. The unequivocal advantage of using the NBD-S-TM probe is its relatively good solubility in water, and for this reason, it is not necessary to use a large amount of organic co-solvent which can interfere with the oxidants. Oxidizing the NBD-S-TM probe with HOCl produces fluorescent 4-thiomorpholine-7-nitrobenzothiadiazole *S*-oxide (NBD-S-TSO) that can be detected and quantified via HPLC analysis. NBD-S-TSO does not undergo further reaction with HOCl. NBD-S-TM exhibits high reactivity toward HOCl (k = 2.6 ± 0.2) × 10^7^ M^−1^s^−1^), high sensitivity (LOD = 60 nM), and excellent selectivity for the recognition of HOCl in cell-free systems. NBD-S-TM is also helpful in monitoring myeloperoxidase-derived HOCl. All these data underscore the usefulness of the probe for HOCl detection in biological systems. Due to the presence of lysosome-locating moiety and according to the published report [67], we anticipate that the described probe can be applied for detecting lysosomal HOCl. This probe opens opportunities for establishing new experimental paradigms and accelerating mechanistically focused biological discoveries. We expect that NBD-S-TM could be an effective tool to discern the physiological and pathological functions of HOCl.

## Figures and Tables

**Table 1:** Comparison of photophysical properties of nitrobenzodiazoles derivatives compounds in a phosphate buffer at pH 7.4.

Compound	λ_abs_ (nm) (ε (10^3^ M^−1^cm^−1^))	λ_em_ (nm) (Φ_em_ (%))	λ_exc_ (nm)	Stokes Shift (nm)
NBD-S-TM ^1^	490 (26.3), 365 (7)	550 (1.00)	490	60
NBD-TM ^2^	350 (7.8), 500 (22)	550 (0.2)	500	50
NBD-Se-TM ^3^	345, 520 (18)	620 (0.06)	520	100
NBD-S-TSO ^1^	475 (22.2)	550 (3.35)	475	75
NBD-TSO ^2^	485 (23.3)	550 (2.4)	485	65
NBD-Se-TSO ^3^	500 (16.7)	620 (0.49)	500	120

^1^ Fluorescein as standard from Ref. [47]; ^2^ from Ref. [35]; ^3^ from Ref. [36].

**Table 2:** Comparison of nitrobenzodiazole-based fluorescent probes for hypochlorous acid.

	R	Probes	Detection Limit (nM)	Response Time (M^−1^s^−1^)	Ref.
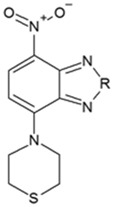	O	NBD-TM	72	1.0 × 10^7^	[35]
	Se	NBD-Se-TM	258	2.0 × 10^7^	[36]
	S	NBD-S-TM	60	2.6 × 10^7^	This work

## Data Availability

Not applicable.

## Supplementary Materials

The following supporting information can be downloaded at https://www.mdpi.com/article/10.3390/molecules28166055/s1. The document provides additional data, including synthesis protocols, ^1^H NMR, ^13^C NMR, HRMS ESI spectra, determination of quantum yield, detection limit, and stability in the presence of H_2_S [35,36,47,68,69,70,71,72].

## Sample Availability

Samples of all synthesized compounds are available from the authors.
