# Supplementary material for: A New and Fast-Response Fluorescent Probe for Monitoring Hypochlorous Acid Derived from Myeloperoxidase
Source: Molecules. 2023 Aug 14;28(16):6055. doi: 10.3390/molecules28166055 (PMC10459737; doi:10.3390/molecules28166055)
Supplement: Supplementary file 1 [file molecules-28-06055-s001.zip › molecules-2547873-supplementary.pdf]

Contents:

1.1. Synthesis ... 1
1.2. Determination of quantum yield ... 2
1.3. Determination of detection limit. ... 3
1.4. Stability in the presence of H_2S ... 4
1.5. ^{1}H NMR, ^{13}C NMR and ESI MS spectra ... 5
1.6. Comparison of fluorescent probes based on HOCl response ... 11
References ... 11

1.1. Synthesis

To a solution of 7-chloro-4-nitrobenzo-2,1,3-thiadiazole (NBD-S-Cl) (0.5 mmol) in DCM (5 mL), thiomorpholine or its *S*-oxide (0.5 mmol) and Et_3N (0.6 mmol) were added. The mixture was stirred at a room temperature for 20 h under argon atmosphere. Next, the solvent was evaporated under reduced pressure, and the residue was dissolved in DCM (30 mL). The organic phase was washed with a citric acid solution (20% aq.), distilled water and the saturated $NaHCO_3$ solution. Finally, the organic phase was dried over anhydrous Na_2SO_4, and the solvent was removed in vacuo. The residue was further purified on silica column chromatography using DCM:MeOH (9.5:0.5) as eluent to yield the solid product.

4-Thiomorpholino-7-nitrobenzothiadiazole (NBD-S-TM) was obtained in 85% yield as an orange powder. ^{1}H NMR (DMSO-d$_6$:CDCl$_3$, 4:1, 600 MHz) δ (ppm): 8.56 (d, J = 9.0 Hz, 1H), 6.88 (d, J= 9.0 Hz, 1H), 4.42-4.43 (m, 4H), 2.84-2.86 (m, 4H); ^{13}C NMR (151 MHz): δ (ppm): 149.6; 147.8; 147.4; 132.5; 128.6; 106.6; 52.7; 27.0

HRMS (ESI): m/z 283.0330 ([M$^+$H]$^+$), Calcd for (M $^+$ H)$^+$ = 283.3420; HRMS (ESI-Na) m/z 305.0150 ([M$^+$Na]$^+$), Calcd for (M$^+$ Na $^+$ H)$^+$ = 305.3312.

***4-Thiomorpholino-7-nitrobenzenothiadiazole S*-oxide** (NBD-S-TSO) was obtained in 50% yield as a dark orange powder.^{1}H NMR (DMSO-d$_6$:CDCl$_3$, 4:1, 600 MHz) δ (ppm): 8.59 (d, J = 9.0 Hz, 1H), 7.00 (d, J= 9.0 Hz, 1H), 4.81 (d, J = 15 Hz 2H), 4.33 (t, J = 12.6 Hz, 2H), 3.11-3.16 (m, 2H), 2.90-2.92 (m, 2H); ^{13}C NMR (151 MHz): δ (ppm): 149.5; 147.5; 132.5; 129.1; 128.9; 106.8; 45.3; 41.4

HRMS (ESI): m/z 299.0277 ([M$^+$H]$^+$), Calcd for (M $^+$ H)$^+$ = 299.3414; HRMS (ESI-Na) m/z 321.0091 ([M$^+$Na]$^+$), Calcd for (M$^+$ Na $^+$ H)$^+$ = 321.3306.

1.2. Determination of quantum yield.

Fluorescence quantum yields were determined by the comparative method using the procedure described previously [S1]. Fluorescence quantum yields were determined using solutions of Quinine sulfate (ΦF = 0.79 in 0.1 M NaOH) as a standard [S2]. The quantum yield was calculated using the following equation:

$$\Phi^F{}_x = \Phi^F{}_s(A_sF_x/A_xF_s)(n_x/n_s)^2$$

- Φ^F is the fluorescence quantum yield;
- A is the absorbance at the excitation wavelength;
- F is the area under the corrected emission curve;

- n is the refractive index of the solvents used;
- subscripts s and x refer to the standard and unknown samples, respectively.

1.3. Determination of detection limit.

The limit of detection (LOD) is the lowest signal and was calculated from the titration curve of the NBD-S-T fluorescence in the presence of HOCl and the mean blank. The fluorescence intensity of NBD-S-TM was measured and the standard deviation of the blank measurements was obtained and determined as σ using the following equation:

$$\sigma = \sqrt{\frac{\sum(\bar{x} - Xi)^2}{n - 1}}$$

- σ is the standard deviation of the blank;
- x is the blank mean;
- Xi is the values of the blank measures;
- n is the blank number tested (n = 11).

Detection limit LOD is calculated using the following equation

Detection limit = 3.3σ/s

- s is the slope of fluorescence intensity versus HOCl concentrations.

1.4. Stability in the presence of H_2S

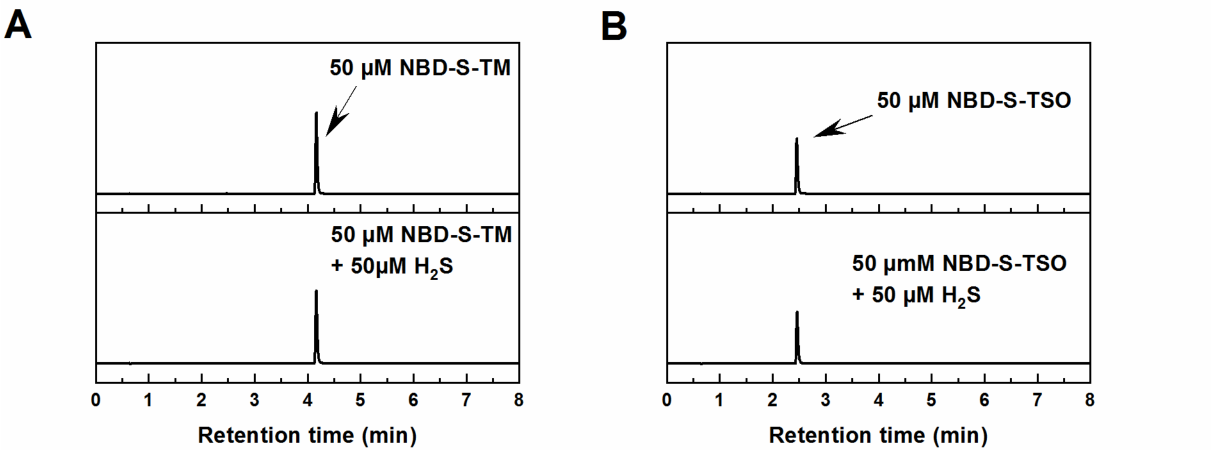

Figure S1. (**A**) The NBD-S-TM probe and (**B**) NBD-S-TSO standard stability in the presence of H_2S. All experiments were carried out in an aqueous solution containing phosphate buffer (50 mM, pH 7.4) and MeCN (10%) at room temperature. The traces were collected using an absorption detector set at 500 nm.

1.5. ^{1}H NMR, ^{13}C NMR and ESI MS spectra

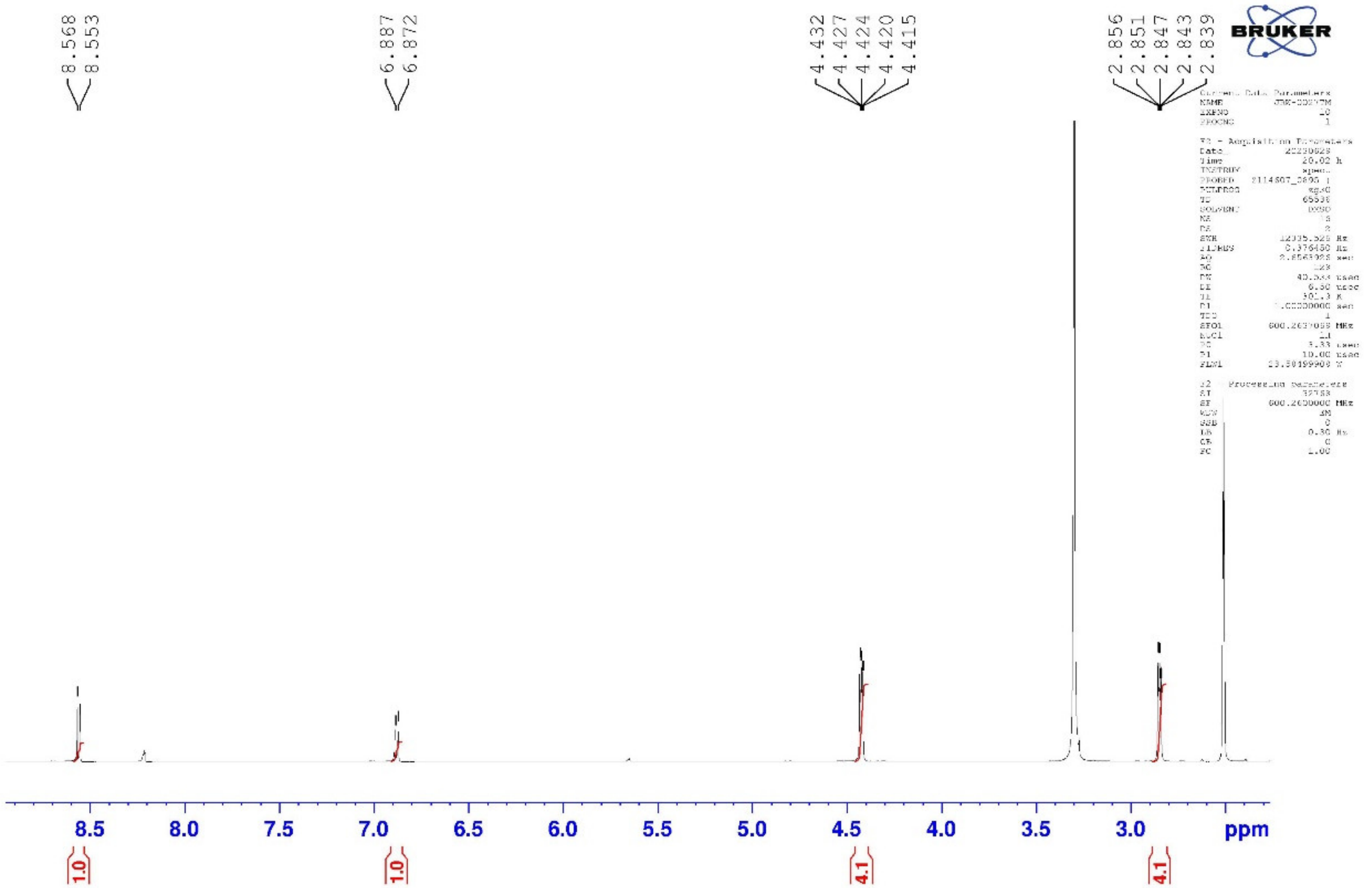

Figure S2. ^{1}H NMR spectra of NBD-S-TM.

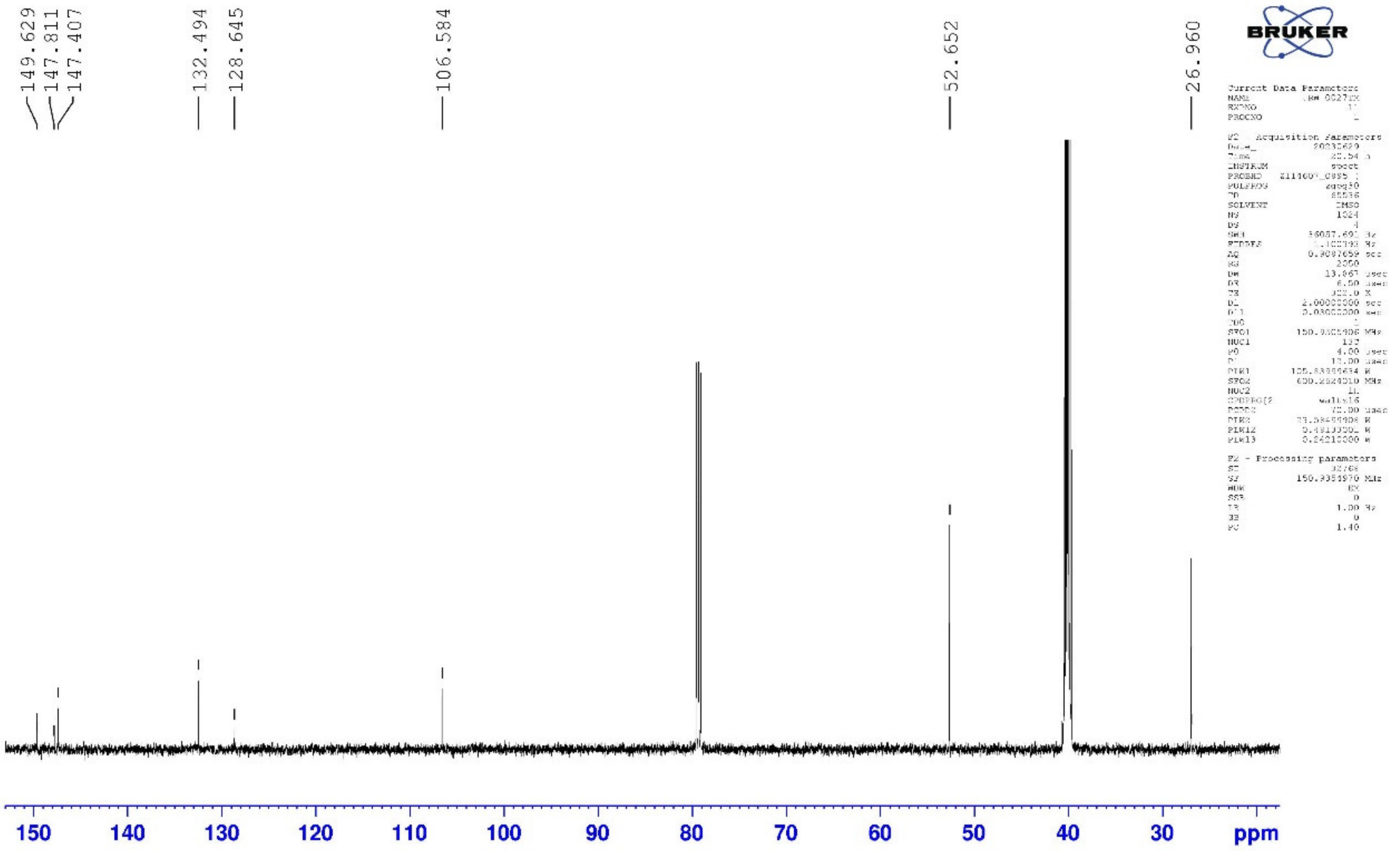

Figure S3. ^{13}C NMR spectra of NBD-S-TM.

Elemental Composition Report

Page 1

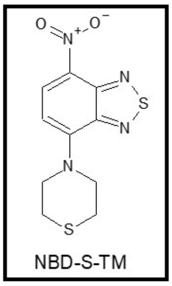

Single Mass Analysis

Tolerance = 5.0 PPM / DBE: min = -1.5, max = 80.0
Element prediction: Off
Number of isotope peaks used for i-FIT = 9

Monoisotopic Mass, Even Electron Ions
75 formula(e) evaluated with 1 results within limits (all results (up to 1000) for each mass)
Elements Used:
C: 0-15 H: 0-30 N: 0-4 O: 0-3 S: 1-2

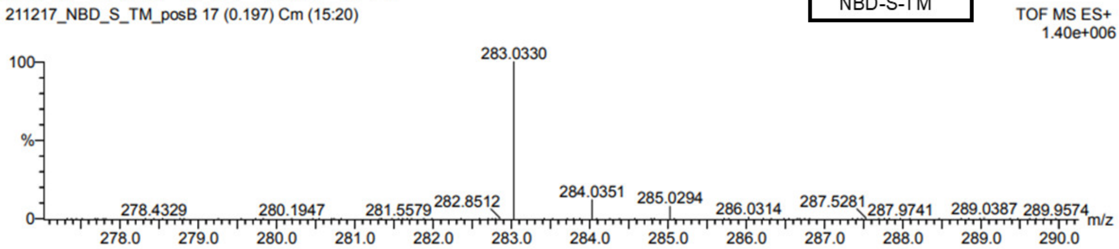

Minimum:				-1.5				
Maximum:		5.0	5.0	80.0				
Mass	Calc. Mass	mDa	PPM	DBE	i-FIT	Norm	Conf(%)	Formula
283.0330	283.0323	0.7	2.5	7.5	400.1	n/a	n/a	C10 H11 N4 O2 S2

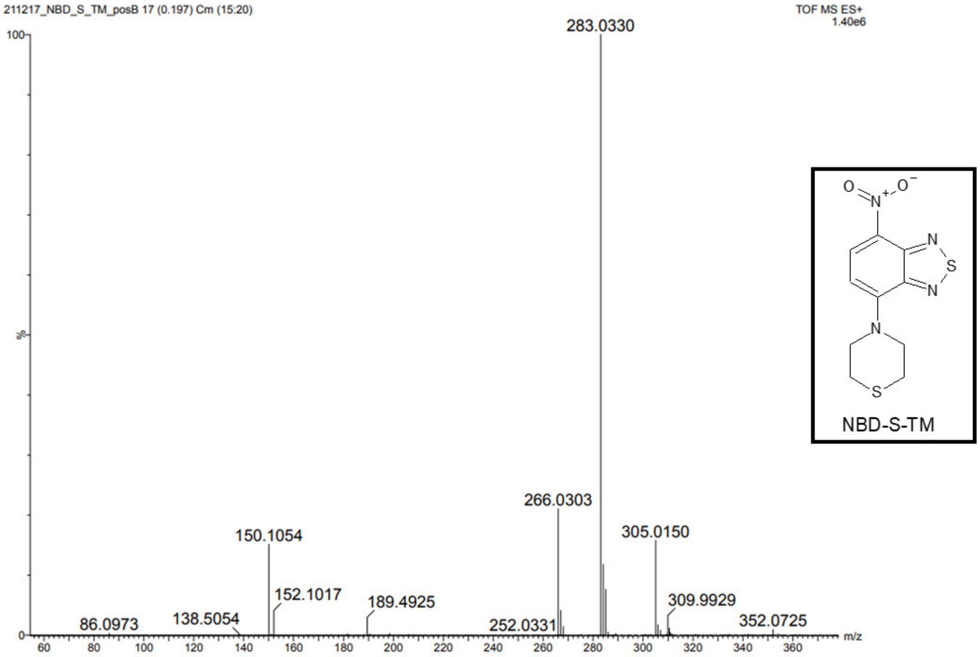

Figure S4. ESI MS spectra of NBD-S-TM.

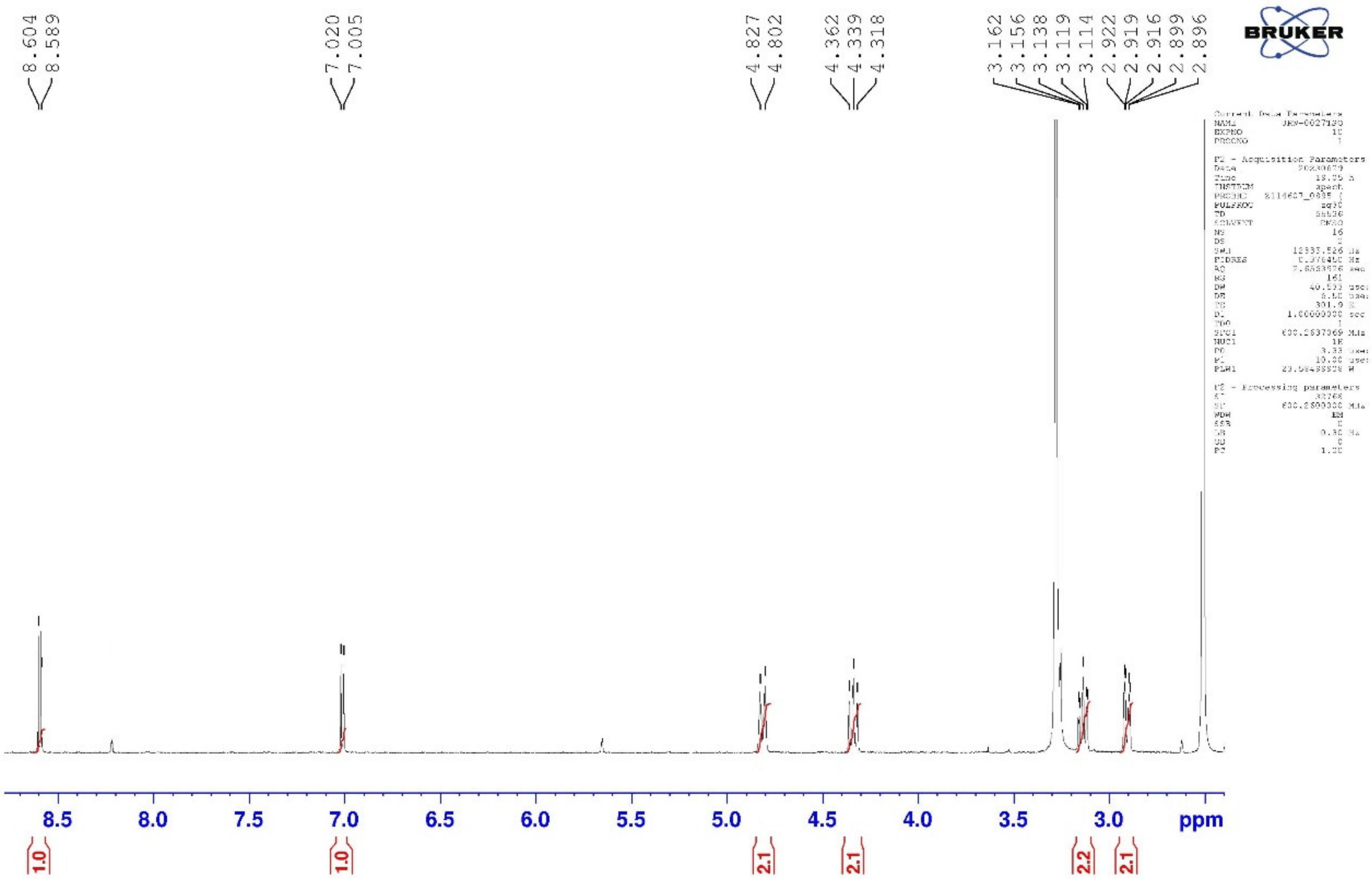

Figure S5. ^{1}H NMR spectra of NBD-S-TSO.

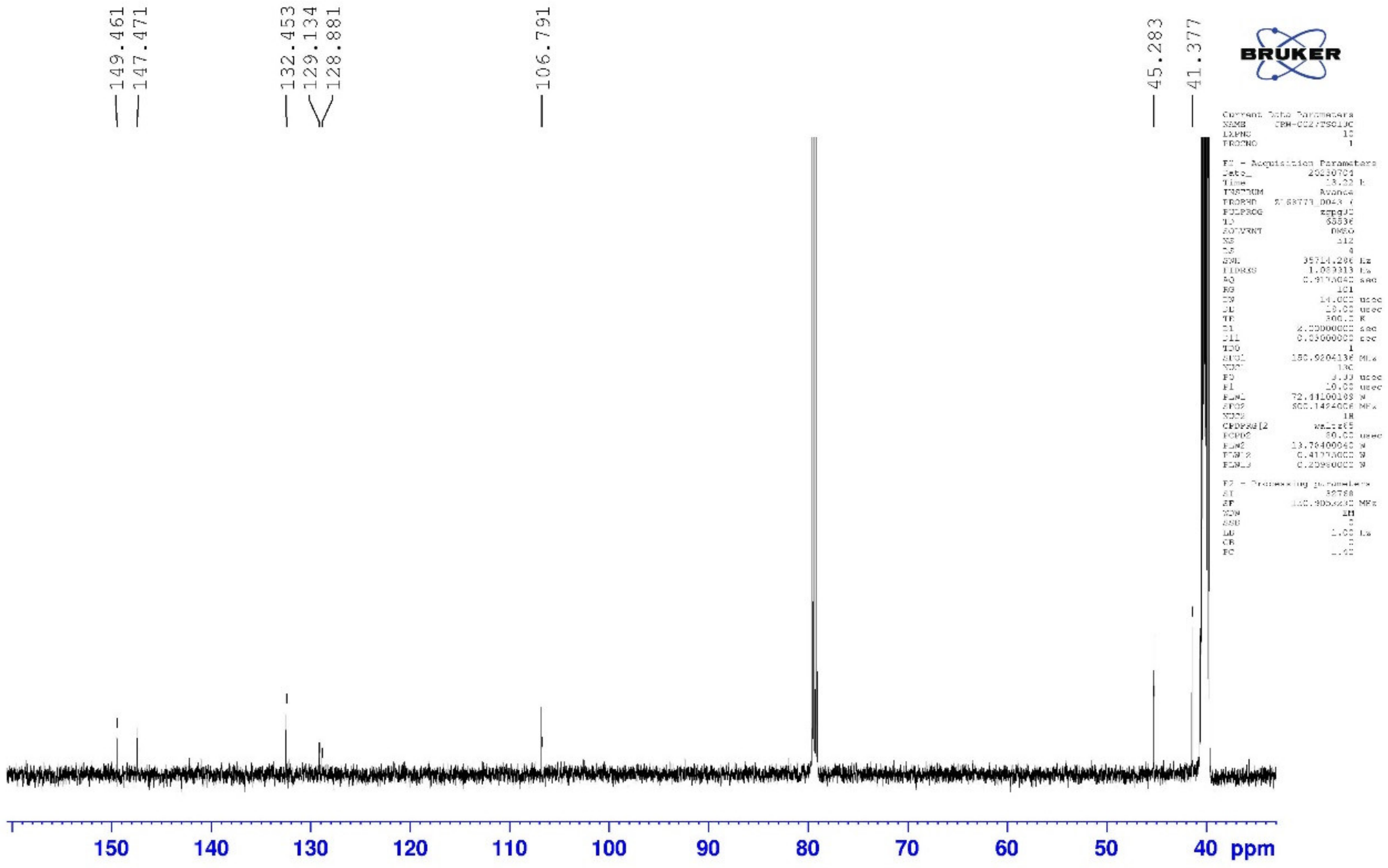

Figure S6. ^{13}C NMR spectra of NBD-S-TSO.

Elemental Composition Report

Page 1

Single Mass Analysis

Tolerance = 5.0 PPM / DBE: min = -1.5, max = 80.0
Element prediction: Off
Number of isotope peaks used for i-FIT = 9

Monoisotopic Mass, Even Electron Ions
61 formula(e) evaluated with 1 results within limits (all results (up to 1000) for each mass)
Elements Used:
C: 0-15 H: 0-30 N: 0-4 O: 0-3 S: 1-2

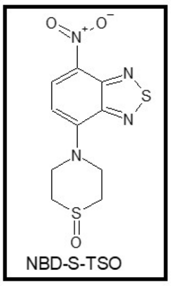

211217_NBD_S_TSO_posB 17 (0.197) Cm (15:20-(4:8+47:52))

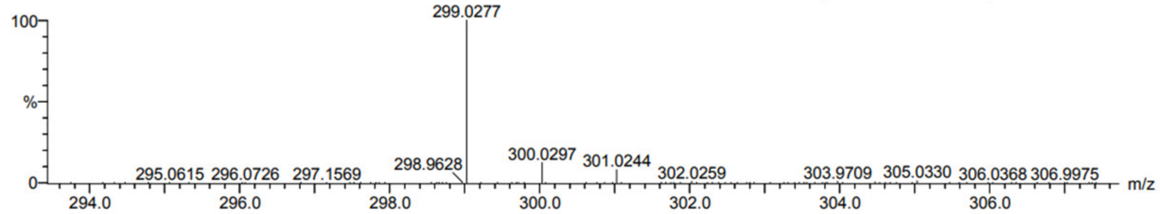

Minimum:				-1.5				
Maximum:		5.0	5.0	80.0				
Mass	Calc. Mass	mDa	PPM	DBE	i-FIT	Norm	Conf(%)	Formula
299.0277	299.0273	0.4	1.3	7.5	593.4	n/a	n/a	C10 H11 N4 O3 S2

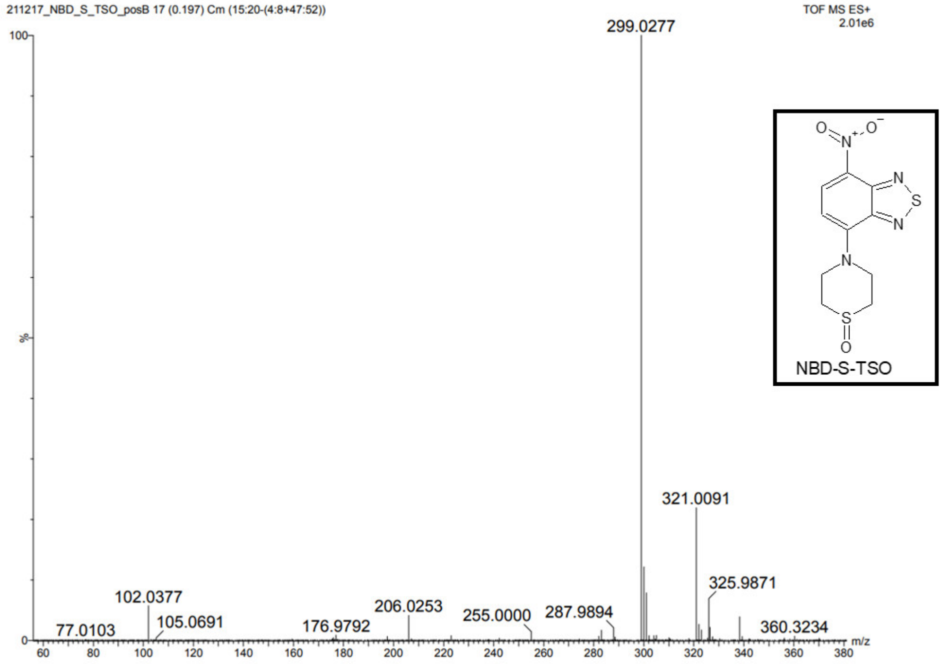

Figure S7. ESI MS spectra of NBD-S-TSO.

1.6. Comparison of fluorescent probes based on HOCl response

Table S1. Comparison of fluorescent probes based on HOCl response.

Probe	LOD	Response time	Mechanism	References
BCy-S	35.2 nM	less than 40 s	TICT	[3]
Probe 1	72 nM	30 s	ICT	[4]
FNIR-HOCl	70 nM	less than 20 s	-	[5]
NBD-TM	72 nM	within 1 s	PET	[1]
NBD-Se-TM	258 nM	within 1 s	PET	[6]
HBTN	24.5 nM	less than 20 min	-	[7]
PTA	33.9 nM	45 s	ICT	[8]
NBD-S-TM	60 nM	within 1 s	PET	This work

References

S1. Świerczyńska, M.; Słowiński, D.; Grzelakowska, A.; Szala, M.; Romański, J.; Pierzchała, K.; Siarkiewicz, P.; Michalski, R.; Podsiadły, R. Selective, Stoichiometric and Fast-Response Fluorescent Probe Based on 7-Nitrobenz-2-Oxa-1,3-Diazole Fluorophore for Hypochlorous Acid Detection. *Dyes and Pigments* **2021**, *193*, 109563, doi:10.1016/j.dyepig.2021.109563.

S2. Umberger, J.Q.; LaMer, V.K. The Kinetics of Diffusion Controlled Molecular and Ionic Reactions in Solution as Determined by Measurements of the Quenching of Fluorescence1,2. *J. Am. Chem. Soc.* **1945**, *67*, 1099–1109, doi:10.1021/ja01223a023.

S3. Shao, S.; Yang, T.; Han, Y. A TICT-Based Fluorescent Probe for Hypochlorous Acid and Its Application to Cellular and Zebrafish Imaging. *Sensors and Actuators B: Chemical* **2023**, *392*, 134041, doi:10.1016/j.snb.2023.134041.

S4. Zheng, Y.; Wu, S.; Bing, Y.; Li, H.; Liu, X.; Li, W.; Zou, X.; Qu, Z. A Simple ICT-Based Fluorescent Probe for HOCl and Bioimaging Applications. *Biosensors* **2023**, *13*, 744, doi:10.3390/bios13070744.

S5. Jiang, J.; Wang, S.; Wang, S.; Yang, Y.; Zhang, X.; Wang, W.; Zhu, X.; Fang, M.; Xu, Y. In Vivo Bioimaging and Detection of Endogenous Hypochlorous Acid in Lysosome Using a Near-Infrared Fluorescent Probe. *Anal. Methods* **2023**, *15*, 3188–3195, doi:10.1039/D3AY00338H.

S6. Świerczyńska, M.; Słowiński, D.; Michalski, R.; Romański, J.; Podsiadły, R. A Thiomorpholine-Based Fluorescent Probe for the Far-Red Hypochlorous Acid Monitoring. *Spectrochimica Acta Part A: Molecular and Biomolecular Spectroscopy* **2023**, *289*, 122193, doi:10.1016/j.saa.2022.122193.

S7. Qu, W.; Yang, B.; Guo, T.; Tian, R.; Qiu, S.; Chen, X.; Geng, Z.; Wang, Z. A Dual-Response Mitochondria-Targeted NIR Fluorescent Probe with Large Stokes Shift for Monitoring Viscosity and HOCl in Living Cells and Zebrafish. *Analyst* **2023**, *148*, 38–46, doi:10.1039/D2AN01693A.

S8. Shang, Z.; Yang, X.; Meng, Q.; Tian, S.; Zhang, Z. A Ratiometric Near-Infrared Fluorescent Probe for the Detection and Monitoring of Hypochlorous Acid in Rheumatoid Arthritis Model and Real Water Samples. *Smart Molecules n/a*, e20220007, doi:10.1002/smo.20220007.
